# An optimized mode of interferon intermittent therapy help improve HBsAg disappearance in chronic hepatitis B patients

**DOI:** 10.3389/fmicb.2022.960589

**Published:** 2022-08-30

**Authors:** Minghui Li, Si Xie, Xiaoyue Bi, Fangfang Sun, Zhan Zeng, Wen Deng, Tingting Jiang, Yanjie Lin, Liu Yang, Yao Lu, Lu Zhang, Wei Yi, Yao Xie

**Affiliations:** ^1^Department of Hepatology Division 2, Beijing Ditan Hospital, Capital Medical University, Beijing, China; ^2^Department of Hepatology Division 2, Peking University Ditan Teaching Hospital, Beijing, China; ^3^Division of Hepatology, Hepato-Pancreato-Biliary Center, School of Clinical Medicine, Beijing Tsinghua Changgung Hospital, Tsinghua University, Beijing, China; ^4^Department of Gynecology and Obstetrics, Beijing Ditan Hospital, Capital Medical University, Beijing, China

**Keywords:** intermittent therapy, clinical cure, HBsAg, HBV, interferon

## Abstract

**Background:**

To investigate the effect of intermittent interferon therapy mode on the disappearance of hepatitis B surface antigen (HBsAg) in chronic hepatitis B (CHB) patients.

**Methods:**

This is a retrospective cohort study in CHB patients who were suspended from pegylated interferon α (PEG-IFNα) therapy due to a plateau in HBsAg decline during the initial treatment period, and resumed interferon therapy after an interval of 3–6 months. Patients received entecavir or tenofovir during the interval period. Hepatitis B virus (HBV) virological and serological indexes, clinical biochemical indexes, and blood routine tests were performed at the baseline and every 3 months during follow-up of initial interferon treatment. A functional cure was analyzed as a primary outcome.

**Results:**

A total of 304 patients treated with intermittent PEG-IFNα were included in the statistical analysis, including 215 men and 89 women, aged 37.97 ± 8.53 years, and 73 hepatitis B e antigen (HBeAg)-negative and 231 HBeAg positive patients. In total 59 patients (19.41%) achieved HBsAg disappearance through the initial, intermittent, and retreatment of PEG-IFNα treatment, of whom 43 patients (14.14%) achieved HBsAg seroconversion. Early HBsAg response to initial treatment was significantly associated with HBsAg response at 12 and 24 weeks of retreatment. After the intermission period, the incidence of HBsAg disappearance in patients with early HBsAg response in the retreatment period was 43.87%. The baseline HBsAg and 12-week HBsAg response in the retreatment period had higher predictive value than the initial treatment HBsAg response.

**Conclusion:**

The initial, intermittent, and retreatment mode of interferon can help to improve the HBsAg disappearance rate in CHB patients.

**Clinical trial registration:**

[www.ClinicalTrials.gov], identifier [NCT04028856].

## Introduction

Chronic hepatitis B virus (HBV) infection is the most important factor leading to hepatocellular carcinoma (HCC) in China (Wang et al., 2017; Qin et al., 2019). The disappearance of HBsAg is a crucial indicator for predicting good long-term outcomes. For patients who have achieved disappearance of HBsAg, the 5-year cumulative incidence of HCC is less than 1.0% (Yip et al., 2019). HBV DNA negative, HBeAg and HBsAg seroconverted patients have the strongest immune control against HBV infection and lower incidence of HCC (van Campenhout and Janssen, 2015). Therefore, it is very important to achieve HBsAg disappearance in CHB patients in consideration of the natural annual disappearance rate of HBsAg in those patients is only 0.5–1.5% (Yeo et al., 2019).

Although nucleoside analog (NA) can inhibit HBV DNA replication very effectively, the annual incidence of HBsAg disappearance is still less than 1.0% (Kim et al., 2014). The disappearance of HBsAg brought by interferon (IFN) therapy is an ideal target and focus of clinical research. Our previous studies have shown that the incidence of HBsAg disappearance remains at about 15% in patients treated with interferon antiviral therapy, although various optimization schemes are adopted, including combined therapy and prolonged therapy. The early HBsAg response to interferon therapy can predict the disappearance of HBsAg, and prolonged treatment can increase the incidence of HBsAg disappearance (Li et al., 2017a,b, 2019; Li et al., 2021a; Pan et al., 2021). However, not all patients with early HBsAg response can achieve HBsAg disappearance through prolonged treatment. Once the patient’s HBsAg level reaches a plateau during treatment, the subsequent treatment cannot further reduce HBsAg levels (Li et al., 2021a). In a study in which NAs-treated patients switched to PEG-IFNα therapy, the 96-week course of treatment did not significantly improve the rate of HBsAg loss compared with the 48-week standard course of treatment (20.7 vs. 14.4%, *p* > 0.05) (Hu et al., 2018). It is worth exploring whether there are any other optimized interferon treatment regimens that can improve the disappearance rate of HBsAg in CHB patients. The purpose of this study is to investigate the effect of intermittent interferon therapy on improving the disappearance of HBsAg in patients with CHB.

## Materials and methods

### Patients

**FIGURE 1:**
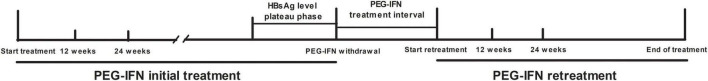
Patient treatment and observation procedures.

CHB patients were from the observation cohort who received PEG-IFNα 180 μg/week in the Second Department of Hepatology, Ditan Hospital from January 2010 to December 2019. During the initial treatment period, the interferon treatment was suspended due to the decline of HBsAg reaching a plateau and resumed again after a 3–6 months interval (Figure 1).

This study was approved by the Ethics Committee of Beijing Ditan Hospital Affiliated with Capital University of Medical Sciences (Jing Di Lun Ke Zi 2018 no. 023-01) and was registered with Clinical Trials (NCT04028856). All authors had access to the study data and reviewed and approved the final manuscript.

### Inclusion and exclusion criteria

Patient inclusion criteria: (1) Aged 18–65 years; (2) Duration of HBsAg-positive more than 6 months; (3) Patient received two stages of PEG-IFNα treatment; (4) Plateau of HBsAg level decline appeared before the withdrawal of interferon treatment. The definition of plateau period: HBsAg level decrease less than 0.5 log IU/mL compared with the previous time point; (5) After the interval of PEG-IFNα treatment, the patients received PEG-IFNα treatment again.

**FIGURE 2:**
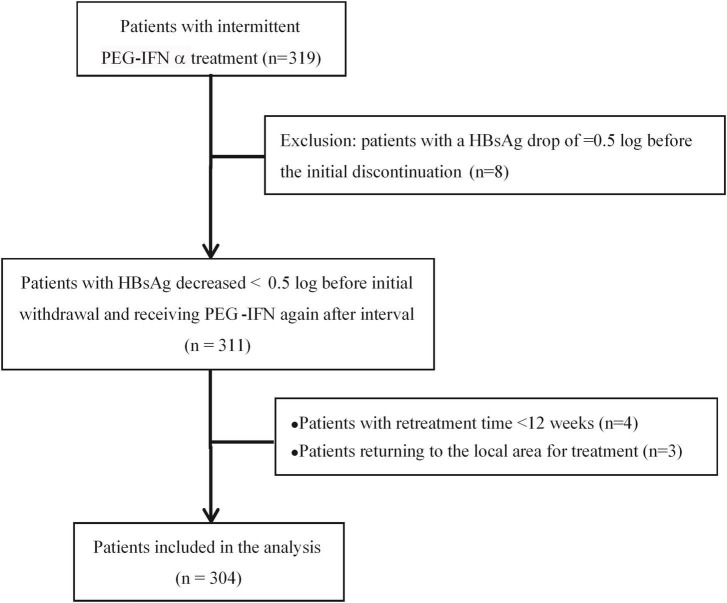
Patient enrollment process.

Exclusion criteria: (1) With other viral infection (HCV, HDV, HEV, HIV); (2) Autoimmune liver disease; (3) Long-term alcoholism and/or the use of other liver damaging drugs; (4) Mental illness; (5) Evidence of liver tumors (liver cancer or AFP > 100 ng/ml); (6) Decompensated liver cirrhosis; (7) Serious diseases of the heart, brain, lung, kidney and other systemic diseases; (8) Used the drugs having Immunosuppressive effect. The patient enrollment process is shown in Figure 2.

### Treatment strategy and grouping

The patients received a subcutaneous injection of PEG-IFNα 180 μg/week in both phases of PEG-IFNα treatment. When the HBsAg level reached a plateau during the first phase of interferon treatment, the interferon treatment was stopped and entered into an intermittent period of 3–6 months. Entecavir (0.5 mg, once daily) or tenofovir (300 mg, once daily) was applied to inhibit virus replication during the interval. After the intermittent period, PEG-IFNα was added for the second stage of interferon retreatment. Entecavir (0.5 mg, once daily) or tenofovir (300 mg, once daily) was applied in the intermittent period, and PEG-IFNα retreatment. After the interval, PEG-IFN was added to NA for combined therapy. For patients who obtained HBsAg disappearance after the combination therapy, drugs were discontinued after 12–24 weeks of consolidation. For patients without HBsAg disappearance, PEG-IFN was stopped while NA maintenance therapy was continued. HBV virological and serological indexes, clinical biochemical indexes, and blood routine tests were performed every 3 months during the initial treatment baseline and treatment follow-up, and liver ultrasound was performed every 3–6 months.

According to the disappearance of HBsAg within 72 weeks of PEG-IFNα retreatment, the patients were divided into HBsAg disappearance group and non-HBsAg disappearance group. HBsAg < 0.05 IU/mL was defined as HBsAg disappeared, and HBsAb ≥ 10 mIU/L was defined as positive. HBsAg response was defined as a ≥ 0.5 log decrease in HBsAg from the baseline during a phase of treatment. HBsAg early response was defined as the 24-week HBsAg level decrease ≥ 0.5 log from the baseline in this phase of treatment.

In general, the setting up of a control group (patients receiving continuous interferon therapy after HBsAg dropped to a plateau during interferon treatment) can better reflect the effect of intermittent therapy on patients whose HBsAg dropped to a plateau during treatment and get high HBsAg loss. However, according to the Guidelines for the Prevention and Treatment of Chronic Hepatitis B (2015) in China, PEG-IFN treatment is recommended to stop for patients with HBV DNA decline of less than 2 Log after 24 weeks of treatment (Chinese Society of Hepatology, 2015). In 2019, China’s Guidelines for the Prevention and Treatment of Chronic Hepatitis B suggested that PEG-IFN treatment should be discontinued for patients with HBsAg decline of less than 1 Log after 24 weeks of treatment (Chinese Society of Hepatology, 2019). Our previous research also found that for patients whose HBsAg decline reached a plateau during PEG-IFN treatment, it was difficult for them to achieve sustained HBsAg decline even after continued treatment (Li et al., 2021b; Lin et al., 2022). Therefore, in view of patients with HBsAg decline entering a plateau cannot benefit if interferon therapy is continued, so we did not set up a control group in this study.

### Detection of clinical indicators

HBV DNA load was detected by CobasTaqMan96 real-time quantitative PCR detection reagent (detection of off-line 20 IU/mL) (Roche, Pleasanton, CA, United States). HBsAg, HBsAb, and HBeAg were detected using Abbott Architect i2000 kits (Abbott Laboratories, Abbott Park, IL, United States). Serum HBsAg levels were determined by Abbott Architect HBsAg QT assay. HBsAg < 0.05 IU/mL was defined as HBsAg disappeared, and HBsAb ≥ 10 mIU/L was defined as positive. Biochemical indexes and peripheral blood count were detected by an automatic biochemical analyzer and automatic blood count analyzer.

### Evaluation indicators

The primary outcome was the incidence of HBsAg disappearance within 72 weeks of interferon retreatment.

### Statistical analysis

Categorical variables were expressed as percentages. Chi-square tests and non-parametric tests were used for comparison between groups. The normal distribution continuous variables were represented by mean ± SD, and the non-normal distribution continuous variables were represented by median and inter-quartile ranges (median, Q1-Q3). The comparison between groups was performed by Student’s *t*-test, analysis of variance, and Kruskal Wallis test. The univariate and multivariate logistic regression analyses were used to analyze the factors associated with HBsAg loss.

## Results

### Patient deposition and features

According to the inclusion criteria, we enrolled 319 patients who received intermittent PEG-IFNα treatment, but 8 patients were excluded for the HBsAg decrease ≥ 0.5 log before the initial treatment discontinuation. Then 311 patients suspended PEG-IFNα therapy when their HBsAg levels reached a plateau and resumed the second stage of additional PEG-IFNα treatment after the intermittent phase of 3–6 months. With 4 patients excluded as the interferon retreatment time was <12 weeks and 3 patients returned to local hospital treatment, 304 eligible patients were included in the final statistical analysis, including 215 men and 89 women, aged 37.97 ± 8.53 years. There were 73 HBeAg-negative patients and 231 HBeAg-positive patients (Figure 2).

### HBsAg appearance during interferon treatment and intermittent

**FIGURE 3:**
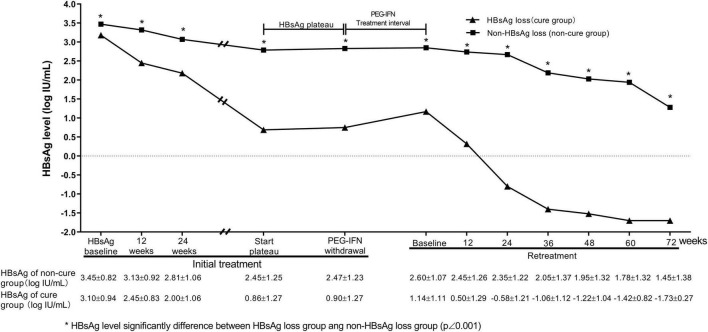
Dynamic changes of HBsAg levels during treatment. There was HBsAg plateau in groups of patients with and without HBsAg loss. Compared with non-HBsAg loss group, HBsAg loss group had a rapid HBsAg decrease at early treatment in the initial and retreatment of PEG-IFNα.

The patients were divided into HBsAg disappearance group (*n* = 59) and non-HBsAg disappearance group (*n* = 245) according to whether HBsAg disappearance was obtained within 72 weeks of retreatment. In the first stage of interferon treatment, the HBsAg levels in both groups first continued to decrease, and then entered a plateau phase. However, the HBsAg levels of the HBsAg-disappeared group were significantly lower than those of the non-HBsAg-disappeared group (*P* < 0.001) at baseline, 12, 24 weeks of treatment in the initial treatment period, and at the withdrawal time point (*P* < 0.001, Figure 3).

During the intermittent period, the HBsAg level in the HBsAg disappearance group was slightly increased after the drug withdrawal. During the interferon retreatment period, the levels of HBsAg in the HBsAg disappearance group and the non-disappearance group decreased at each time point. The HBsAg levels of the HBsAg disappearance group at each time point were significantly lower than those of the non-disappearance group, and the HBsAg drop in the HBsAg disappearance group at 24 weeks during the retreatment period was significantly greater than that of the non-HBsAg disappearance group (1.67 ± 1.07 vs. 0.26 ± 0.40, *t* = 13.717, *P* < 0.001, Figure 3).

### Early HBsAg response during two phases of interferon treatment

In the first stage of interferon treatment, 22.36 and 40.46% of patients had HBsAg response at 12 and 24 weeks, respectively. In the second stage of interferon treatment, 14.7 and 32.23% obtained HBsAg response at 12 and 24 weeks of treatment, respectively (Table 1). There was a significant correlation between HBsAg response at 12 weeks of treatment in the first stage and HBsAg response at 12 weeks of retreatment (Spearman *r* = 0.262, *P* < 0.001) and 24 weeks of retreatment (*r* = 0.252, *P* = 0.001). There was also a significant correlation between HBsAg response at 24 weeks of treatment in the first stage and HBsAg response at 12 and 24 weeks of retreatment [(Spearman *r* = 0.149, *P* = 0.048) vs. (Spearman *r* = 0.328, *P*<0.001)]. Compared with patients without early HBsAg response in the first stage of treatment, patients with early HBsAg response in the first stage had a higher percentage of early HBsAg response in the second stage [20.2% (26/129) vs. 48.0% (59/123), χ^2^ = 21.789, *P*<0.001].

**TABLE 1:** Comparison of characteristics of patients with HBsAg loss and non-HBsAg loss after interferon retreatment.

Index	All patients (*n* = 304)	Non-HBsAg loss (*n* = 245)	HBsAg loss (*n* = 59)	χ^2^ or *t*	*P*
Age (years) (mean ± *SD*)	37.97 ± 83	38.05 ± 8.76	37.81 ± 7.76	0.140	0.889
Man (*n*, %)	215 (70.72%)	172 (70.20%)	43 (72.88%)	0.165	0.685
Baseline HBsAg at initial treatment (Log 10 IU/mL) (mean ± *SD*)	3.37 ± 0.85	3.44 ± 0.81	3.09 ± 0.94	2.855	0.005
Patient of HBeAg positive (*n*, %)	231 (75.98%)	190 (77.55%)	41 (69.49%)	1.693	0.193
HBsAg decline at 12 weeks of initial treatment (Log 10 IU/mL) [median (IQR)]	0.18 (−0.00, 0.52)	0.14 (−0.02, 0.48)	0.31 (0.07, 1.02)	–2.295	0.025
Patient achieved HBsAg decline ≥ 0.5 log IU/mL at 12 weeks of initial treatment (*n*, %)	68 (22.36%)	48 (19.6%)	20 (33.9%)	5.604	0.018
HBsAg decline at 24 weeks of initial treatment (Log 10 IU/mL) [Median (IQR)]	0.45 (0.13, 1.16)	0.37 (0.06, 0.91)	0.96 (0.34, 1.94)	–3.864	<0.001
Patient achieved HBsAg decline ≥ 0.5 log IU/mL at 24 weeks of initial treatment (*n*, %)	123 (40.46%)	88 (35.9%)	35 (59.3%)	10.811	0.001
Duration of initial treatment (weeks) [median (IQR)]	60.00 (48.00, 96.00)	60.00 (41.50, 84.00)	84.00 (48.00, 120.00)	–2.490	0.013
HBsAg decline during initial treatment (Log 10 IU/mL) [median (IQR)]	0.80 (0.27, 1.94)	0.63 (0.15, 1.52)	2.18 (1.01, 3.21)	6.225	<0.001
Patient achieved HBsAg decline ≥ 0.5 log IU/mL during initial treatment (*n*, %)	187 (61.51%)	135 (55.1%)	52 (88.1%)	21.971	<0.001
HBsAg level at the point time before first withdrawal (Log 10 IU/mL) [median (IQR)]	2.43 (1.00, 3.36)	2.78 (1.55, 3.49)	0.69 (−0.10, 1.96)	–8.750	<0.001
Duration of HBsAg descending plateau (weeks) (mean ± *SD*)	14.89 ± 5.50	14.82 ± 5.51	15.18 ± 5.52	0.446	0.656
HBsAg level at first withdrawal (Log 10 IU/mL) [Median (IQR)]	2.50 (1.05, 3.34)	2.83 (1.61, 3.47)	0.74 (0.05, 2.02)	8.774	<0.001
Patients reached undetectable HBV DNA at first withdrawal (*n*, %)	234 (76.97%)	186 (75.91%)	48 (81.35%)	0.793	0.373
Treatment interval (weeks) [median (IQR)]	17.78 (14.00, 31.00)	18.00 (14.00, 31.00)	17.00 (14.00, 35.71)	0.035	0.972
Patients had HBsAg elevation during intermittent treatment (*n*, %)	180 (59.21%)	144 (58.77%)	36 (61.01%)	0.099	0.753
Increased levels of HBsAg during intermittent treatment (Log 10 IU/mL) [median (IQR)]	0.25 (0.09, 0.57)	0.22 (0.08, 0.56)	0.38 (0.13, 0.79)	–2.390	0.018
Baseline HBsAg level at retreatment (Log 10 IU/mL)	2.49 (1.49, 3.32)	2.84 (1.77, 3.47)	1.16 (0.38, 2.05)	9.168	<0.001
HBsAg decline at 12 weeks of retreatment (Log 10 IU/mL) [median (IQR)]	0.06 (−0.07, 0.28)	0.04 (−0.09, 0.18)	0.48 (−0.03, 1.41)	6.666	<0.001
Patient with HBsAg decreased ≥ 0.5 log IU/mL at 12 weeks of retreatment (*n*, %)	44 (14.47%)	22 (9.0%)	20 (33.9%)	24.797	<0.001
HBsAg decline at 24 weeks of retreatment (Log 10 IU/mL) [median (IQR)]	0.24 (0.03, 0.74)	0.14 (0.00, 0.50)	1.53 (0.74, 2.48)	13.717	<0.001
Patient with HBsAg decreased ≥ 0.5 log IU/mL at 24 weeks of retreatment (*n*, %)	98 (32.23%)	55 (22.4%)	43 (72.9%)	20.522	<0.001
Patient reached undetectable HBV DNA at retreatment 24 weeks (*n*, %)	293 (96.06%)	234 (95.51%)	59 (100%)	2.748	0.097
Patient with ALT increased within retreatment 12 weeks (*n*, %)	179 (58.88%)	137 (55.91%)	39 (66.10%)	2.023	0.155
Product of HBsAg declines at 12 weeks of initial treatment and at retreatment 12 weeks [median (IQR)]	0.00 (−0.01, 0.06)	0.00 (−0.01, 0.05)	0.06 (−0.01, 0.74)	3.705	<0.001
Product of HBsAg declines at 24 weeks of initial treatment and at retreatment 24 weeks [median (IQR)]	0.09 (0.00, 0.64)	0.03 (0.00, 0.22)	1.18 (0.27, 2.39)	6.864	<0.001

ALT, Alanine aminotransferase; HBeAg, hepatitis B e antigen; HBsAg, hepatitis B surface antigen.

### Relevant factors affecting occurrence of HBsAg disappearance

After the first stage of treatment and intermission, a total of 59 patients (19.41%) obtained HBsAg disappearance in the second stage of treatment, of whom 43 cases (14.14%) had HBsAg seroconversion. The decreased level of HBsAg in patients with the disappearance of HBsAg in the first stage of interferon treatment was significantly higher than that in patients without the disappearance of HBsAg [2.18 (1.01, 3.21) vs. 0.63 (0.15, 1.52), *t* = 6.225, *P* < 0.001]. Compared with the non-HBsAg disappearance group, the HBsAg disappearance group had lower baseline HBsAg level in the first stage treatment (3.44 ± 0.81 vs. 3.09 ± 0.94, *t* = 2.855, *P* = 0.005) and baseline HBsAg level in the second stage treatment (2.84 [1.77, 3.47] vs. 1.16 [0.38, 2.05], *t* = 9.168, *P* < 0.001). Patients with disappearance of HBsAg had higher early response rate of HBsAg in both stages of treatment (35.9 vs. 59.3%, χ^2^ = 10.811, *P* = 0.001; 22.4 vs. 72.9%, χ^2^ = 20.522, *P* < 0.001, Table 1).

**FIGURE 4:**
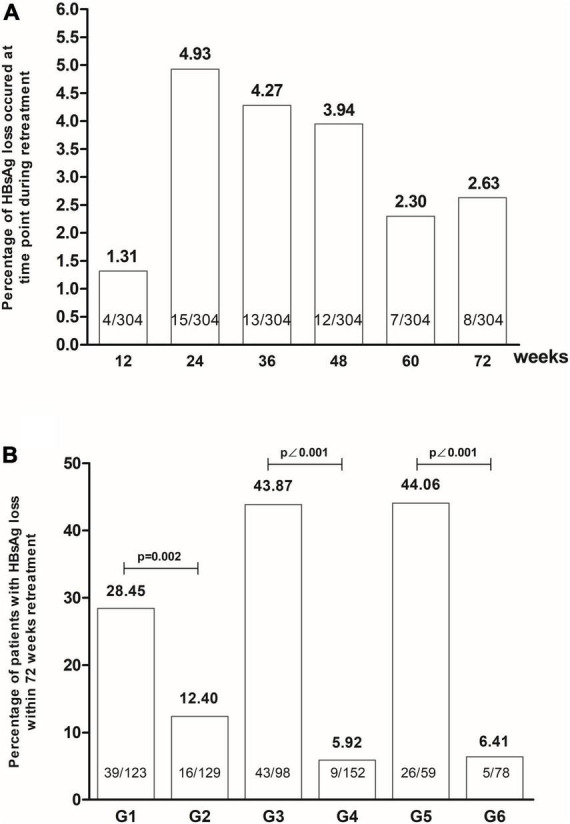
The HBsAg loss occurred at the time of retreatment **(A)**, and the patients with early HBsAg response had high rates of HBsAg loss by retreatment **(B)** (G1: Group with early HBsAg response at initial treatment. G2: Group without early HBsAg response at initial treatment. G3: Group with early HBsAg response at retreatment. G4: Group without early HBsAg response at retreatment. G5: Group with early HBsAg response at initial treatment and at retreatment. G6: Group without early HBsAg response initial treatment and at retreatment).

HBsAg disappeared in 4 (1.31%), 15 (4.93%), 13 (4.27%), 12 (3.94%), 7 (2.30%) and 8 (2.63%) cases during < 12, 12–24, 24–36, 36–48, 48–60 and 60–72 weeks in the second stage of treatment, respectively. Higher HBsAg disappearance rate appeared in patients with early response to HBsAg in the initial treatment compared to non-early responders, in patients with early response to HBsAg in the retreatment compared to patients without it, and in patients with early response to HBsAg in both initial and retreatment compared to patients with no early response at all [28.45% (35/123) vs. 12.40% (16/129), *P* = 0.002; 43.87 (43/98) vs. 5.92% (9/152), *P* < 0.001; 44.06% (26/59) vs. 6.41% (5/78), *P* < 0.001, Figure 4]. Patients with early HBsAg response in retreatment had a higher HBsAg disappearance rate than patients with early HBsAg response in initial treatment [43.87% (43/98) vs. 28.45% (35/123), χ^2^ = 5.681, *P* = 0.017]. There was no significant difference in the disappearance rate of HBsAg between patients with early HBsAg response in retreatment, and patients with early HBsAg response in initial treatment and retreatment [44.06% (26/59) vs. 43.87% (43/98), Fisher, *P* = 1.000].

Univariate logistic analysis showed that the baseline HBsAg level before the first stage of treatment, the early HBsAg response of the first stage of treatment, the HBsAg level during the withdrawal of the first stage of treatment, the length of the first stage of treatment, and the decline of HBsAg during the first stage of treatment were relevant factors for the disappearance of HBsAg after retreatment. The baseline HBsAg level of retreatment and the early HBsAg response of retreatment were also significantly related factors for patients to obtain the disappearance of HBsAg, but there is no significant correlation between the length of time HBsAg levels were at plateau before the withdrawal of initial treatment and the interval time from withdrawal to retreatment, as shown in Table 2.

**TABLE 2:** Logistic regression analysis of related factors of HBsAg disappearance.

Univariate logistic analysis				Multivariate forward stepwise logistic analysis		
	
Index	OR	95% CI	*P*	OR	95% CI	*P*
Age	0.998	0.965–1.032	0.888	/	/	
Man	1.141	0.604–2.154	0.685	**/**	/	
Baseline HBsAg at initial treatment	0.628	0.453–0.872	0.005	/	/	0.641
Patient of HBeAg positive	0.659	0.351–1.238	0.195	/	/	
Patient achieved HBsAg decline ≥ 0.5 log IU/mL at 12 weeks of initial treatment	2.321	1.201–4.486	0.012	/	/	0.884
Patient achieved HBsAg decline ≥ 0.5 log IU/mL at 24 weeks of initial treatment	2.809	1.461–5.401	0.002	/	/	0.593
Duration of initial treatment	1.008	1.001–1.014	0.016	/	/	0.235
HBsAg decline during initial treatment	2.000	1.594–2.511	<0.001	/	/	0.277
HBsAg level at the point time before first withdrawal	0.415	0.322–0.533	<0.001	/	/	0.140
Duration of HBsAg descending plateau	1.011	0.963–1.062	0.655	/	/	/
HBsAg level at first withdrawal	0.409	0.317–0.528	<0.001	/	/	0.167
Patients reached undetectable HBV DNA at first withdrawal	3.613	0.831–15.701	0.087	/	/	/
Treatment interval (weeks)	1.000	0.992–1.008	0.972	/	/	/
Patients had HBsAg elevation during intermittent treatment	1.202	0.663–2.181	0.544	/	/	/
Baseline HBsAg level at retreatment	0.342	0.253–0.463	<0.001	0.376	0.229–0.620	<0.001
Patient with HBsAg decreased ≥ 0.5 log IU/mL at retreatment 12 weeks	6.727	3.131–14.452	<0.001	5.001	1.874–13.344	0.001
Patient with HBsAg decreased ≥ 0.5 log IU/mL at 24 weeks of retreatment	12.422	5.678–27.176	<0.001	/	/	0.104
Patient reached undetectable HBV DNA at retreatment 24 weeks	407320507	0.000–	0.999	/	/	/
Patient with ALT increased within retreatment 12 weeks	1.537	0.848–2.787	0.157	/	/	/

ALT, Alanine aminotransferase; CI, confidence interval; HBeAg, hepatitis B e antigen; HBsAg, hepatitis B surface antigen; OR, odds ratio.

We further conducted a multivariate forward stepwise logistic regression analysis. The results showed that only the baseline HBsAg in the retreatment period (OR 0.376, 95% CI: 0.229–0.620, *p* = 0.001) and the decrease of HBsAg > 0.5 log IU/mL (OR 5.001, 95% CI: 1.874–13.344, *P* = 0.001) at 12 weeks of retreatment were the significant factors for the disappearance of HBsAg during retreatment.

## Discussion

The efficiency of antiviral treatment for CHB is affected by many factors such as HBV, drugs, treatment schemes, and immune cell functions (Wang et al., 2021; Ye and Chen, 2021). In interferon treatment, the clearance half-life of HBV-infected hepatocytes varies greatly in different patients (Sypsa et al., 2005), so 75% of HBsAg disappearance occurs after 48 weeks in extended treatment (Li et al., 2021a). Although these patients who can obtain HBsAg disappearance have a good early response, prolonging the course of treatment is useful to improve the rate of HBsAg disappearance (Li et al., 2017a,b, 2019; Li et al., 2021a; Pan et al., 2021). China’s 2019 Guidelines for The Prevention and Treatment of Chronic Hepatitis B recommended that “the course in patients who achieved good response by 48 weeks treatment can be extended as needed” (Wang et al., 2019). Prolonged treatment is supposed to increase the incidence of HBsAg disappearance, but not all patients with early response can obtain HBsAg disappearance from prolonged treatment (Li et al., 2021a).

In a clinical study of patients with chronic hepatitis C treated with interferon, patients with virologic response had higher expression of IFN receptors in their PBMC before treatment as compared with patients without response. However, IFN receptors expression decreased gradually in the responding patients and was correlated with antiviral treatment response, while PBMC receptors expression did not change in the non-responding patients (Massirer et al., 2004). PEG-IFN α may be limited by its depletion of CD8 T cells *in vivo* (Micco et al., 2013). It was found that there was desensitization of gene expression in fibroblasts induced by interferon stimulation cultured *in vitro*. The gene expression decreased with prolonged interferon stimulation. After stopping the interferon stimulation culture for a period of time, stimulation with interferon again can restore the stimulating effect of interferon on transcriptional gene expression (Larner et al., 1986). When PEG-IFN is used to treat patients with chronic hepatitis B, it brings a direct antiviral effect and enhanced immune cell function. These findings suggested that patients would enter a plateau of HBsAg decline during PEG-IFN treatment for chronic hepatitis B. Previous studies have shown that a decline in HBsAg from baseline of less than 0.5 log IU/mL after 12 weeks of PEG-IFN treatment predicted difficulty in achieving a sustained virological response after discontinuation of treatment (Moucari et al., 2009). In ETV-treated patients adding to PEG-IFN for 12 weeks, HBsAg decreased by > 0.5 log IU/mL after 12 weeks of treatment and decreased by > 1.0 log IU/mL after 24 weeks of treatment can effectively predict HBsAg disappearance after treatment (Li et al., 2015). These studies indicated that for patients without a significant and sustained decline in HBsAg during PEG-IFN treatment it was difficult to obtain a good response to subsequent treatment. Therefore, we took the decrease of HBsAg < 0.5 log IU/mL compared with the previous time point as the leveling value of HbsAg decline plateau.

CHB is clinically cured by interferon treatment. It relies on the combination of interferon and cell surface receptors to initiate the production of intracellular antiviral proteins to directly inhibit viral replication and viral antigen production, and stimulate immune cell activity, achieving the purpose of clearing virus-infected hepatocytes (Cao et al., 2018, 2021; Li et al., 2021b). In some patients with interferon treatment advantages and early responses, the reason for not persisting with subsequent treatment may be related to the down-regulation of interferon receptor expression during interferon therapy (Massirer et al., 2004), and is also related to the depletion of CD8^+^ cytotoxic T cells (CTL) in the presence of viral antigens (Micco et al., 2013). The curative effect cannot be further extended, which means that the viral response enters a plateau and prolonged treatment cannot help HBsAg levels continue to decline. Therefore, in clinical practice, prolonged treatment cannot help all patients with early response reach HBsAg disappearance. These studies revealed part of the reason why the efficacy of interferon antiviral therapy is not sustainable. However, the effectiveness of interferon anti-HBV is affected by multiple factors, and no specific immunological indicator has been identified that can accurately predict the therapeutic response to interferon anti-HBV therapy. In clinical practice, HBV virology or serological indicators and their changes are still used to predict the antiviral efficacy of interferon.

To our knowledge, there are no studies on how to manage patients with CHB if their response indicators entered a plateau during PEG-IFN treatment. We think that after suspended interferon therapy, patients may be retreated with interferon to achieve better outcomes, such as clinical cure. In this study, the effect of interferon intermittent therapy mode was analyzed. In this study, when the HBsAg level reached a plateau during the first-stage interferon treatment, the interferon treatment was stopped. It is assumed that the number of interferon receptors and the function of CTL will be restored through an intermittent period of 3–6 months. In regard to inhibiting virus replication, NAs are stronger than PEG-IFN. In patients maintaining viral response who underwent ETV treatment, if they switched ETV to PEG-IFN alfa-2a therapy, 39.17% of patients had a virological breakthrough or viral rebound (Ning et al., 2014). In a study of optimal therapeutic strategy in NA experienced CHB using peginterferon, in the group switched from ETV to peginterferon treatment, viral relapse after discontinuing PEG-IFNα 24 weeks in 79.6% of patients (Lim et al., 2022). Studies have shown that in patients with naïve treatment, only 17–30% of HBeAg-positive patients and 42–50% of HBeAg-negative patients achieved sustained viral response after 24 weeks of discontinuation of PEG-IFN treatment (Buster et al., 2007; Piratvisuth et al., 2008). However, in NA-treated patients, viral relapse occurred in 79.6% of them after discontinuation of PEG-IFN treatment (Lim et al., 2022). Therefore, all patients in this study were treated with ETV or TDF at intervals to maintain effective inhibition of virus replication. After the interval, both naïve and NA-treated patients were treated with PEG-IFN in addition to NA maintenance therapy. Previous studies have shown that 28.0% of patients who were converted to PEG-IFN monotherapy after NA treatment experienced HBV DNA conversion to positive during PEG-IFN treatment (Ning et al., 2014). The incidence of HBsAg disappearance was higher in patients treated with NA in combination with PEG-IFN than those in PEG-IFN monotherapy (Marcellin et al., 2016). Thus, all patients in this study were treated with NA and PEG-IFN in the retreatment stage. NA can effectively inhibit HBV replication and reduce cccDNA complement, which may be more conducive to the occurrence of HBsAg disappearance. However, the disappearance of HBsAg is mainly attributed to PEG-IFN.

Our study showed that 19.41% of patients achieved HBsAg disappearance and 14.14% achieved HBsAg seroconversion through initial, intermittent, and retreatment of PEG-IFNα. The results show that such a model of interferon intermittent therapy could help improve HBsAg disappearance in CHB patients. In this study, although the retreatment of PEG-IFN combined with ETV or TDF, HBsAg loss should be considered the effect of PEG-IFN. In above mentioned two studies of switching ETV to PEG-IFN, no HBsAg loss was observed in the group continually using NA (Ning et al., 2014; Lim et al., 2022).

In the interferon antiviral therapy of CHB, the early HBsAg response can predict the occurrence of HBsAg disappearance (Li et al., 2017a,b, 2019; Li et al., 2021a; Pan et al., 2021). In this study, 40.46% of patients with initial treatment achieved an early HBsAg response, but they entered an HBsAg plateau during subsequent treatment. The analysis of the correlation between early HBsAg response and HBsAg disappearance showed that although the early HBsAg response in the retreatment period was significantly correlated with the early HBsAg response in the initial treatment, the incidence of HBsAg disappearance in the patients with early HBsAg response in retreatment was significantly higher than that in the initial period. However, the HBsAg disappearance rate of patients with early HBsAg response in both initial treatment and re-treatment was not significantly different from that in patients with early HBsAg response after re-treatment. The results of this study suggest that in the interferon treatment mode of initial, intermittent, and re-treatment, the early HBsAg response in the retreatment period is more valuable in predicting HBsAg disappearance than the HBsAg early response in the initial treatment.

In this study, 28.45% of patients with early HBsAg response in the initial treatment period achieved HBsAg disappearance through retreatment. Multivariate logistic regression showed that baseline HBsAg during the retreatment period and HBsAg response at 12 weeks of retreatment were independent predictors of HBsAg disappearance obtained in the initial interferon treatment, intermittent, and retreatment modes. The results of the study indicate that some patients whose HBsAg cannot be continuously decreased in the initial treatment may achieve HBsAg disappearance through intermittent treatment. The baseline HBsAg and 12-week HBsAg response in the re-treatment period have higher predictive values than the initial treatment HBsAg response.

In our study, inter-group comparative analysis and single-factor logistic regression analysis showed that some indicators were significantly correlated with the occurrence of HBsAg disappearance. However, multi-factor logistic regression analysis showed that only the baseline level of HBsAg on retreatment and the decrease of HBsAg > 0.5 logIU/mL after 12-week retreatment were the related factors for the occurrence of HBsAg loss. All patients in this study underwent intermittent and PEG-IFN retreatment due to the plateau phase of HBsAg decline during the initial phase. The HBsAg baseline of retreatment was affected by the baseline level and initial treatment. Therefore, in the prediction analysis of HBsAg disappearance in the overall treatment plan, it is difficult to obtain the cutoff value for predicting HBsAg disappearance in the overall treatment plan by ROC curve analysis with the baseline HBsAg values from retreatment alone. Our study suggests that it is worthwhile to re-treat patients after suspension of interferon therapy in patients who had good initial treatment response and achieved low levels of HBsAg but entered a plateau of HBsAg decline.

Strategies to pursue clinical cure of CHB include a selection of advantageous patients, prediction of curative effect based on early response, combination therapy and prolonged therapy, etc. (Li et al., 2017a,b, 2019; Li et al., 2021a; Pan et al., 2021). For patients with both an advantage in baseline HBsAg level and an early HBsAg response, interferon therapy is often suspended once the efficacy of the patient’s treatment cannot be continued. Considering that the loss of efficacy may be caused by the down-regulation of interferon receptor expression and the depletion of CD8 + T cells, resulting in decreased sensitivity to interferon (Massirer et al., 2004; Micco et al., 2013), we may optimize the treatment strategies to restore patients’ sensitivity to interferon therapy to achieve HBsAg disappearance, through suspending interferon therapy and then resuming treatment after a period of intermittent period.

There are some limitations of our study, such as the inability to obtain HBV genotypes because these tests were not routinely performed before treatment. Of course, most of the HBV strains circulating in China are HBV genotype B or C. No baseline-matched continuous treatment control cohort was established, and the effect of virus-related and other factors on changes in HBsAg during treatment (including HBV DNA load, genotype, family history, and medication history) was not analyzed.

## Conclusion

In conclusion, the initial, intermittent, and retreatment mode of interferon is helpful for improving the HBsAg disappearance rate in CHB patients, supporting that more patients can obtain functional cures through the optimized treatment.

## Data availability statement

The raw data supporting the conclusions of this article will be made available by the authors, without undue reservation.

## Ethics statement

This study was approved by the Ethics Committee of Beijing Ditan Hospital Affiliated to Capital University of Medical Sciences (Jing Di Lun Ke Zi 2018 No. 023-01) and was registered with Clinical Trials (NCT04028856). Written informed consent for participation was not required for this study in accordance with the national legislation and the institutional requirements.

## Author contributions

ML, WY, and YX contributed to the study design. ML, SX, XB, and FS contributed to the data analysis. ML, SX, XB, FS, YoL, LZ, and YX contributed to the recruitment, enrollment, and assessment of participants, as well as data collection. FS, WD, TJ, and XB contributed to the following up with the patients. ZZ, YnL, and LY managed all aspects of laboratory support. ML wrote the first draft of the manuscript. YX revised the manuscript and is the guarantor of the article. All authors approved the final version of the manuscript.

## Abbreviations

ALT: alanine aminotransferase
CHB: chronic hepatitis B
CI: confidence interval
CTL: cytotoxic T cells
ETV: entecavir
HBeAg: hepatitis B e antigen
HBsAg: hepatitis B surface antigen
HBV: hepatitis B virus
HCC: hepatocellular carcinoma
HCV: hepatitis C virus
HDV: hepatitis D virus
HEV: hepatitis E virus
human immunodeficiency virus: HIV
IFN: interferon
NA: nucleoside analog
OR: odds ratio
PEG-IFN α: pegylated interferon α
TDF: tenofovir disoproxil fumarate.
